# Departure from multiplicative interaction for catechol-O-methyltransferase genotype and active/passive exposure to tobacco smoke among women with breast cancer

**DOI:** 10.1186/1477-3163-5-3

**Published:** 2006-01-17

**Authors:** Brian D Bradbury, Jemma B Wilk, Ann Aschengrau, Timothy L Lash

**Affiliations:** 1 Department of Epidemiology, Amgen Inc., 1 Amgen Center Drive MS: 24-2-A, Thousand Oaks, CA 91320, USA; 2 Deparment of Neurology, Boston University School of Medicine, 715 Albany St. B601, Boston, MA 02118, USA; 3 Department of Epidemiology, Boston University School of Public Health, 715 Albany St. T3E, Boston, MA 02118, USA; 4 Geriatrics Section, Department of Medicine, Boston University School of Medicine, 715 Albany St. B2, Boston, MA 02118, USA

## Abstract

**Background:**

Women with homozygous polymorphic alleles of catechol-O-methyltransferase (COMT-LL) metabolize 2-hydroxylated estradiol, a suspected anticarcinogenic metabolite of estrogen, at a four-fold lower rate than women with no polymorphic alleles (COMT-HH) or heterozygous women (COMT-HL). We hypothesized that COMT-LL women exposed actively or passively to tobacco smoke would have higher exposure to 2-hydroxylated estradiol than never-active/never passive exposed women, and should therefore have a lower risk of breast cancer than women exposed to tobacco smoke or with higher COMT activity.

**Methods:**

We used a case-only design to evaluate departure from multiplicative interaction between COMT genotype and smoking status. We identified 502 cases of invasive incident breast cancer and characterized COMT genotype. Information on tobacco use and other potential breast cancer risk factors were obtained by structured interviews.

**Results:**

We observed moderate departure from multiplicative interaction for COMT-HL genotype and history of ever-active smoking (adjusted odds ratio [aOR] = 1.6, 95% confidence interval [CI]: 0.7, 3.8) and more pronounced departure for women who smoked 40 or more years (aOR = 2.3, 95% CI: 0.8, 7.0). We observed considerable departure from multiplicative interaction for COMT-HL genotype and history of ever-passive smoking (aOR = 2.0, 95% CI: 0.8, 5.2) or for having lived with a smoker after age 20 (aOR = 2.8, 95% CI: 0.8, 10).

**Conclusion:**

With greater control over potential misclassification errors and a large case-only population, we found evidence to support an interaction between COMT genotype and tobacco smoke exposure in breast cancer etiology.

## Background

A major focus in breast cancer research has been the carcinogenic role of estrogens. In addition to investigating breast cancer risk factors that influence lifetime exposure to estrogen (*e.g*. age at menarche and age at menopause) [1,2], research is beginning to examine the environmental and genetic factors that affect estrogen metabolism [3]. It is known that naturally occurring estrogen (17β-estradiol) is hydroxylated at different sites by cytochrome P450 enzymes to form catechol estrogens including 2-hydroxylated (30–40%) and 16α-hydroxlated catechol estrogen (10–12%) [4]. 16α-hydroxylated catechol estrogen has been implicated in cell transformation and genotoxic damage [5,6] whereas 2-hydroxylated catechol estrogen has been postulated as a potentially anti-carcinogenic estrogen metabolite [7-9]. Both metabolites compete with estrogen for the estrogen receptor protein, but only the 16α-hydroxylated form has estrogenic activity [10-12]. Michnovicz *et al*. [13] found that active cigarette smoking results in a substantial increase in 2-hydroxylation but little change in 16α-hydroxylation, contributing to a higher ratio of 2-hydroxlyated/16α-hydroxylated estradiol. Findings from recent studies support the hypothesis that a higher 2-/16α-metabolite ratio is protective against breast cancer development [14-17].

The 2-hydroxylated catechol estrogen is metabolized by catechol-O-methyltransferase (COMT) into non-genotoxic methyl ethers that are excreted from the body. There are two COMT alleles, the wild-type allele is related to high activity (COMT-H), and the polymorphic allele (COMT-L) in its homozygous form is related to an approximately four-fold lower activity [18,19]. The COMT-L allele results from a single nucleotide polymorphism in the COMT gene, a G → A mutation at codon 158, which leads to a substitution of methionine for valine [20]. Individuals homozygous for the polymorphism (COMT-LL) have low COMT activity, individuals homozygous for the wild-type allele (COMT-HH) have high COMT activity, and heterozygous individuals (COMT-HL) have intermediate COMT activity [20]. Thus, the COMT alleles have a codominant (or additive) effect on metabolic activity level

This variability in methyltransferase activity related to the polymorphism has prompted research investigating the association between COMT genotype and breast cancer risk. The epidemiologic studies that have examined the association have produced inconsistent results [21-37]. A few studies have found an elevated breast cancer risk for women with low COMT activity [21-26]. Others have reported no association between COMT activity and breast cancer risk [25-35], and a few have found a decreased breast cancer risk for women with low COMT activity [32,37]. A recent meta-analysis concluded that there was no major association between COMT polymorphisms and breast cancer risk [32].

Four earlier studies have examined the association between COMT activity and the risk of breast cancer within sub-groups of tobacco exposure [21,24,25,28]. Two found that the elevated breast cancer risk for women with low COMT activity was greater among non-smokers than smokers [21,24] and two found no meaningful difference in risk of breast cancer by COMT activity across smoking groups [25,28].

We collected genetic and behavioral information on 502 cases of incident breast cancer arising in five different sites across the United States. We used a case-only design to examine the potential interaction between methylation status – as measured by COMT genotype – and active or passive smoking status among women with breast cancer. We hypothesized that women who have low COMT activity and are exposed to tobacco smoke should have an enriched concentration of 2-hydroxylated catechol estrogen, and should therefore have a lower smoking-related risk of breast cancer than women with high COMT activity. The case-only design is optimal for assessing departure from multiplicative interaction when the genotype and environmental exposure are independent of one another. This investigation is the largest case-only study to date to examine the interaction between COMT genotype and tobacco exposure history as it relates to breast cancer.

## Methods

### Study population

The cases of female breast cancer included in this analysis were identified as parts of two study populations [38,39]. The first population includes cases of invasive breast cancer diagnosed between 1987 and 1993 among residents of eight towns in Cape Cod, Massachusetts, and who were reported to the Massachusetts Cancer Registry. The second population includes cases of pathologically confirmed, stage I or stage II breast cancer that were diagnosed from December 1996 to September 1999 at hospitals in Rhode Island, North Carolina, Minnesota, or Los Angeles, California. The study was conducted with the approval of the Boston University Medical Campus Institutional Review Board.

### Data collection

#### Buccal cell samples for genotyping

Breast cancer cases were mailed introductory letters in 2001 and 2002. A trained interviewer followed the letter with a telephone call to answer questions and solicit participation. Cases who agreed to participate were sent an enrollment package containing an introductory letter, summary information about the study, a consent form, instructions for submitting a mouthwash sample, a safety-sealed sample of mouthwash, and a wide-mouth sample collection bottle. Participants collected the sample and returned it in a postage-paid box along with their informed consent. Buccal cells were precipitated by centrifugation and stored at -70°C until a batch of 90 samples had been collected. Batches were sent by overnight delivery on dry ice to Qiagen Genomics (Bothell, WA) for DNA extraction and genotyping.

Qiagen genomics applied proprietary Masscode technology to measure Masscode tags, which are low molecular weight compounds linked to the DNA via a photocleavable linker. The tag is cleaved in flow into a mass spectrometer, and a Microsoft Access database converts the raw analytical data into statistically generated genotype calls. The assay has been validated in over one million genotypes. Existing primers were used to characterize COMT genotypes at the codon 158 single nucleotide polymorphism (SNP) in each buccal cell sample. The Qiagen genotyping data characterized each participant as homozygous wild-type, heterozygous, or homozygous polymorphic at the SNP.

#### Interview data

Cases were interviewed on the telephone by trained interviewers using a structured interview to obtain information on demographic characteristics, history of active and passive exposure to cigarette smoke, and known or suspected risk factors for breast cancer. Cases from the Cape Cod population were interviewed between March 1997 and March 1998. Cases from the second population were interviewed approximately forty months after their date of diagnosis to gather the variables used in this analysis. Most of the exposure information related to early life characteristics and events, so the differential delay between diagnosis and interview should have little impact on the results.

### Analytic variables

#### COMT Genotype

We considered a woman a fast methylator if she was homozygous for the COMT-H wild-type allele (COMT-HH), an intermediate methylator if she was a heterozygote (COMT-HL, carrying both the COMT-H wild-type allele and the COMT-L polymorphic allele), and a slow methylator (COMT-LL) if she was homozygous for the COMT-L polymorphic allele. Women who were COMT-LL were the reference genotype for all analyses.

#### Tobacco exposure

We considered a woman an active smoker if she reported smoking 100 or more cigarettes in her lifetime. We considered a woman a passive smoker if she reported living with someone who was a smoker but was never herself a smoker. For women who reported smoking 100 or more cigarettes in their lifetime or who lived with someone who smoked, information on duration, intensity and timing of exposure to tobacco smoke (active/passive) was also collected. Women who were neither active nor passive smokers were labeled never active/never passive smokers and were the tobacco exposure reference group for all analyses.

#### Covariates

In addition to smoking information, we collected information on health and behavioral risk factors including alcohol use, body mass index (BMI), family history of breast cancer, history of benign breast disease, and parity. BMI was calculated as weight divided by the square of height (kg/m^2^). A woman was considered to have a first-degree family history of breast cancer (yes/no) if she reported that her mother, sister(s) or daughter(s) was diagnosed with breast cancer. We defined alcohol use according to the number of drinks a woman reported that she "usually" had before her diagnosis: non-drinker, ≤ one drink/month, few drinks/month, few drinks/week, almost every day, and unknown.

### Analytic strategy

We examined departure from multiplicative interaction for the association between methylation status and exposure to cigarette smoke (gene-environment interaction) among women with breast cancer. We used logistic regression analysis in SAS (Cary, NC) to quantify departure from multiplicative interaction. In these analyses, the odds ratios (OR) and 95% confidence intervals (CI) for the association between smoking status and methylation status estimate the departure of the gene and environment joint effect from multiplicative interaction. We examined the associations separately for active and passive smokers. We assessed the interaction between tobacco exposure and COMT genotype separately for high (COMT-HH) and intermediate COMT (COMT-HL) activity as compared to low COMT activity (COMT-LL). Women classified as both COMT-LL and never active/never passive smokers were the reference category for all analyses. We controlled for the potential influence of breast cancer risk factors including age at breast cancer diagnosis, alcohol consumption, BMI, first-degree family history of breast cancer, geographic location (state where breast cancer diagnosis was made, which also controls for study sample), and history of benign breast disease using multiple variable logistic regression. We did not assess the influence of race in the analysis since 97% of the women in our case population were white.

We also evaluated departure from multiplicative interaction for variables describing duration, intensity and timing of active and passive smoking exposure. For active smokers, we examined the association between methylation status and the number of packs of cigarettes smoked per day, duration of smoking (in years), age at onset of smoking, and time since quitting. For passive smokers, we examined the duration of passive exposure (in years) and the age at first passive exposure.

## Results

Among the Cape Cod population, 330 of 483 eligible cases agreed to receive a sample collection kit and the remainder refused or were unable to be contacted. Of the 330 who received a kit, 272 returned a sample and 269 samples yielded DNA that could be genotyped. Among the second study population, 372 of 410 eligible cases agreed to receive a sample collection kit and the remainder refused or were unable to be contacted. Of the 372 who received a kit, 321 returned a sample and 233 had samples that yielded DNA that could be genotyped and had the requisite interview data. In both studies, 56% of eligible participants were genotyped and included in the analysis. The proportion of smokers among non-participants was not significantly different than among participants in either study population. The mean age was greater among non-participants than among participants (mean age 66 versus 61 years in the Cape Cod population, p = 0.0001; mean age 74 versus 73 years in the second study population, p = 0.03), reflecting greater losses-to-follow-up and refusals among older women. Age was not, however, associated with genotype among the participants. Furthermore, the proportions of participants who were COMT-LL, COMT-HL, COMT-HH, active smokers, and passive smokers did not vary significantly with their site of enrollment. Among the genotyped controls in the Cape Cod study, the odds ratio associating methylation status (homozygotes versus heterozygotes) with exposure to tobacco smoke (ever-active smoking versus all others) equaled 1.10 (p = 0.80). This finding is consistent with earlier studies [24,40] in which COMT genotype and active smoking were not significantly associated among controls.

There were 502 cases of breast cancer available for analysis. Eighty women (16%) were COMT-LL, 248 (49%) were COMT-HL and 174 (35%) were COMT-HH. Table 1 provides demographic and risk factor characteristics of the breast cancer cases according to COMT genotype. The distribution of breast cancer risk factors did not differ materially according to COMT genotype.

**Table 1:** Distribution of breast cancer risk factors by catechol-*O*-methyltransferase (COMT) genotype

	COMT-LL (N = 80)		COMT-HL (N = 248)		COMT-HH (N = 174)	
Characteristic	N	%	N	%	N	%

Age						
< 50	8	10	29	13	23	13
50 – 59	5	6	25	10	13	7
60 – 69	28	35	70	28	64	37
70+	39	49	124	50	74	43
Alcohol use						
Non-drinker	12	15	39	16	26	15
≤ 1 drink/month	20	25	51	21	47	27
Few drinks/month	20	25	58	23	38	22
Few drinks/week	12	15	61	25	42	24
Almost every day	14	17	31	13	19	11
Unknown	2	3	8	3	2	1
Body Mass Index (BMI) kg/m^2^						
< 20.0	13	16	45	18	31	18
20.0 – 24.9	55	69	145	59	110	63
25.0 – 29.9	8	10	41	17	25	14
30.0+	2	3	11	4	6	4
Missing	2	3	6	2	2	1
Family History of Breast Cancer *						
No	62	77	188	76	130	75
Yes	17	22	54	22	44	25
Missing	1	1	6	2	0	0
Geography						
Massachusetts	37	46	134	54	98	56
California	10	13	30	12	20	11
Rhode Island	13	16	29	12	13	7
Minnesota	9	11	25	10	28	16
North Carolina	11	14	30	12	15	9
History of Benign Breast Disease						
No	52	65	153	61	117	67
Yes	27	34	94	38	52	30
Missing	1	1	1	1	5	3
Parity						
Nulliparous	14	17	46	18	39	22
1 live birth	7	9	21	8	16	9
2 live births	17	21	39	16	43	25
3 live births	18	23	64	26	36	21
4 live births	13	16	39	16	15	9
5 or more live births	11	14	39	16	25	14

* Includes a woman's mother, sister(s) and/or daughter(s).

We observed moderate departure from multiplicative interaction for COMT-HL genotype and history of ever-active smoking (adjusted odds ratio [aOR] = 1.6, 95% CI: 0.7, 3.8) but minimal departure from multiplicative interaction for COMT-HH genotype and history of ever-active smoking (aOR = 1.2, 95% CI: 0.4, 3.1) compared to COMT-LL genotype (Table 2).

**Table 2:** Crude and adjusted odds ratios (OR) and adjusted 95% confidence intervals for departure from multiplicative interaction between COMT genotype and risk of active exposure to tobacco

	COMT Genotype			COMT-HL vs. COMT-LL	COMT-HH vs. COMT-LL
Measures of dose, duration and timing	COMT-LL	COMT-HL	COMT-HH	aOR*§ (95% CI)	aOR*§ (95% CI)

Never active/never passive ‡	12	32	24	1.0 (--)	1.0 (--)
Active smoker	39	123	85	1.6 (0.7–3.8)	1.2 (0.4–3.1)
Packs (per day)					
< 1 packs	24	75	44	1.5 (0.6–3.9)	1.1 (0.4–3.1)
≥ 1 packs	15	45	40	1.6 (0.6–4.5)	1.2 (0.4–3.7)
Missing	0	3	1	†	†
Duration (years)					
< 40	29	83	60	1.3 (0.5–3.4)	1.1 (0.4–3.0)
40+	9	40	24	2.3 (0.8–7.0)	1.4 (0.4–4.9)
Missing	1	0	1	†	†
Age started (years)					
< 22	30	95	66	1.6 (0.7–4.1)	1.1 (0.4–3.0)
≥ 22	8	28	18	1.5 (0.5–4.6)	1.5 (0.4–5.4)
Missing	1	0	1	†	†
Quit smoking before diagnosis date (years)					
Current/<5	11	17	16	0.8 (0.2–2.5)	0.7 (0.2–2.5)
5 – 15	9	29	18	1.6 (0.5–5.0)	0.8 (0.2–2.9)
> 15	17	57	39	1.5 (0.6–4.2)	1.1 (0.4–3.3)
Missing	2	20	12	†	†

* Adjusted odds ratio

§ Controlling for age, alcohol use, age at first birth, BMI, family history of breast cancer, history of benign breast disease, and geographic site of breast cancer diagnosis

‡ Reference group for all comparisons

† Estimates not provided for category of missing values

Estimates for the departure from multiplicative interaction between COMT genotype and the measures of intensity, duration and timing of active smoking were generally higher for COMT-HL genotype than for COMT-HH genotype. The departure from multiplicative interaction was greater than the null for women with COMT-HL genotype when they smoked one or more packs per day (aOR = 1.6, 95% CI: 0.6, 4.5), smoked for 40 or more years (aOR = 2.3, 95% CI: 0.8, 7.0), or started smoking before age 22 (aOR = 1.6, 95% CI: 0.5, 4.6). The departure from multiplicative interaction was greater than the null for women with COMT-HH genotype when they smoked one or more packs per day (aOR = 1.2, 95% CI: 0.4, 3.7) or smoked for 40 or more years (aOR = 1.4, 95% CI: 0.4, 4.9).

We observed considerable departure from multiplicative interaction for COMT-HL genotype and history of ever-passive smoking (adjusted odds ratio [aOR] = 2.0, 95% CI: 0.8, 5.2) and moderate departure from multiplicative interaction for COMT-HH genotype and history of ever-passive smoking (aOR = 1.6, 95% CI: 0.6, 4.5) compared to COMT-LL genotype (Table 3).

**Table 3:** Crude and adjusted odds ratios (OR) and adjusted 95% confidence intervals for departure from multiplicative interaction between COMT genotype and risk of passive exposure to tobacco

	COMT Genotype			COMT-HL versus COMT-LL	COMT-HH versus COMT-LL
Measures of dose, duration and timing	COMT-LL	COMT-HL	COMT-HH	aOR*§ (95% CI)	aOR*§ (95% CI)

Never active/never passive‡	12	32	24	1.0 (--)	1.0 (--)
Passive smoker	29	93	65	2.0 (0.8–5.2)	1.6 (0.6–4.5)
Duration (years)					
< 40	20	69	47	1.6 (0.6–4.3)	1.1 (0.3–3.3)
40+	8	23	16	5.1 (1.1–23)	5.3 (1.0–28)
Missing	1	1	2	†	†
First lived w/smoker (age in years)					
< 12	12	46	28	2.3 (0.7–7.1)	1.0 (0.3–3.9)
12 – 20	6	11	6	0.7 (0.2–3.2)	1.1 (0.2–6.9)
> 20	9	28	26	2.8 (0.8–10)	3.3 (0.8–13)
Missing	2	8	5	†	†

* Adjusted odds ratio

§ Controlling for age, alcohol use, age at first birth, BMI, family history of breast cancer, history of benign breast disease, and geographic site of breast cancer diagnosis

‡ Reference group for all comparisons

† Estimates not provided for category of missing values

Estimates for the departure from multiplicative interaction between COMT genotype and the measures of intensity, duration and timing of passive smoking were generally of higher magnitude for COMT-HH activity than COMT-HL activity. The departure from multiplicative interaction was greater than the null for women with COMT-HL genotype who were passively exposed to tobacco smoke for 40 or more years (aOR = 5.1, 95% CI: 1.1, 23) and who first lived with a smoker after 20 years of age (aOR = 2.8, 95% CI: 0.8, 10). Similarly, the departure from multiplicative interaction was greater than the null for women with COMT-HH genotype who were passively exposed to tobacco for 40 or more years (aOR = 5.3, 95% CI: 1.0, 28) and who first lived with a smoker after 20 years of age (aOR = 3.3, 95% CI: 0.8, 13).

## Discussion

This is the largest case-only study to evaluate the interaction between catechol-*O*-methyltransferase (COMT) activity and active/passive exposure to cigarette smoke and only the second to remove women passively exposed to tobacco smoke from the reference group, as has been recommended for studies of the relation between tobacco smoke and breast cancer risk [41]. Our analysis found evidence to support a departure from multiplicative interaction between COMT activity and smoking status. The relative risk of breast cancer for women with the COMT-HL genotype and a history of ever-active smoking was 1.6-fold (95% CI: 0.7, 3.8) higher than the product of the relative risks associated with the genotype alone and smoking alone. Similarly, the relative risk of breast cancer for women with the COMT-HL genotype (aOR = 2.0, 95% CI: 0.8, 5.2) or the COMT-HH genotype (aOR = 1.6, 95% CI: 0.6, 4.5) and a history of residential exposure to passive smoke were higher than the product of their respective relative risks associated with the genotypes alone and passive exposure alone.

The analyses assessing the departure from multiplicative interaction between the genotypes and intensity, duration or timing of active and passive exposure to cigarette smoke yielded estimates consistent with a promotional role in breast cancer etiology for exposure to tobacco smoke. The relative risk of breast cancer among women with the COMT-HL or COMT-HH genotype who were actively or passively exposed to tobacco smoke for a long duration was higher than expected from the independent effects of the genotype and smoking exposure. These data are consistent with following hypothesis for the interaction between COMT genotype and exposure to tobacco smoke. First, tobacco smoke exposure increases 2-hydroxylation of 17β-estradiol [13], which leads to a higher ratio of 2-hydroxlyated/16α-hydroxylated estradiol and confers a protective effect against breast cancer development [14-17]. Second, COMT-HH or COMT-HL genotype yields greater capacity to metabolize 2-hydroxylated estradiol, compared with COMT-LL genotype. Our data suggest that women with long-term tobacco smoke exposure and with the COMT-HH or COMT-HL genotype have a higher risk of breast cancer than women with (a) long-term tobacco smoke exposure and the COMT-LL genotype – perhaps because these women have higher circulating concentrations of 2-hydroxylated estradiol due to their low COMT activity, or (b) no tobacco smoke exposure and the COMT-HH or COMT-HL genotypes – perhaps because these women have lower circulating concentrations of 2-hydroxylated estradiol due to the absence of tobacco smoke exposure.

Only a few studies have assessed potential modification by cigarette smoking on the risk of breast cancer for women with COMT-LL as compared to COMT-HH. Lavigne *et al*. [21] found an elevated risk of breast cancer for COMT-LL women compared to COMT-HH women, and this increased risk was greater for non-smokers (OR = 2.8) than smokers (OR = 1.8), suggesting that cigarette smoking attenuates any increased risk of breast cancer for women with slow catechol-O-methyltransferase activity. These findings were based on 114 cases and controls and did not account for the influence of passive exposure to cigarette smoke. Yim *et al*. [24] found that never smokers with COMT-HL and COMT-LL genotypes were at greater breast cancer risk as compared to never smokers with COMT-HH genotype, OR = 2.0 and OR = 1.7, respectively. The two remaining studies found no evidence of an interaction between COMT activity and active [25,28] or passive [28] smoking.

Previous studies of the association between COMT genotype and breast cancer risk have compared women with one or more copies of the polymorphism to a reference group of women homozygous for the wild-type allele [21,24,25,28]. In our study, we used women homozygous for the polymorphism as the reference group to clarify the interpretation of our effect estimates. Below the null departure from multiplicative interaction is difficult to interpret because it may reflect sub-multiplicative interaction that is still super-additive. In contrast, departure from multiplicative interaction above the null reflects positive interaction on both the additive and multiplicative scales.

The genotyping procedures employed in this analysis have superior accuracy compared to PCR-RFLP techniques employed in three of the previous studies [21,24,25]. Consequently, the rates of misclassification of methylation status in this study should be less than rates in the previous studies employing less accurate PCR-RFLP techniques. Misclassification of either variable involved in an assessment of interaction in case-control studies can give rise to the appearance of interaction when, in fact, there is none [42].

Our analysis of interaction using case-only data provides greater control over the impact of potential misclassification errors, because there are only two variables central to the analysis that are susceptible to misclassification – COMT genotype and smoking status. As described above, the impact of misclassification is less predictable in three of the previous case-control analyses. It is therefore possible that previous studies evaluating the interaction of COMT genotype and exposure to tobacco smoke in relation to breast cancer risk may have generated biased estimates of interaction. By using a more accurate genotyping method and by implementing a case-only design, this analysis provides a more valid assessment of departure from multiplicative interaction between COMT genotype and exposure to tobacco smoke in relation to breast cancer.

Weighing against this advantage of the case-only design is the limitation that only departure from multiplicativity can be assessed. Many epidemiologists weigh departure from additive interaction more heavily, arguing that the additive scale corresponds better to the biologic meaning of synergistic effects [43]. A further limitation of the case-only design is its reliance on the assumption that the genetic polymorphisms and environmental exposure are independent of one another [44]. Violations of this assumption can substantially distort the estimates of interaction. However, COMT polymorphisms and smoking history were not associated among the genotyped controls in the Cape Cod study or among the controls in earlier studies [24,40]. The absence of association supports the independence assumption required to validly estimate departure from multiplicativity with the case-only design.

Finally, these results must be interpreted with the following limitation in mind. Only 56% of eligible cases were available for analysis. Participation was not related to smoking status and although participation was related to age, age was not related genotype. We expect that the selection of participants introduced no substantial bias, although we acknowledge that our study of breast cancer survivors may have influenced the estimates of effect in ways that we are unable to anticipate.

## Conclusion

This large case-only analysis is only the second to investigate the interaction between COMT genotype and exposure to tobacco smoke as related to breast cancer risk while removing women passively exposed to tobacco smoke from the reference group. The combination of the most complete genotyping data and the large case-only design provided important advantages in this study, whose results suggest potential interaction between COMT genotype and tobacco smoke exposure in breast cancer etiology, perhaps related to the coaction of smoking and genotype on the concentration of 2-hydroxylated catechol estrogen. Weighing against this interpretation is the potential for an unanticipated bias to have arisen by selection of breast cancer survivors from among the incident cases.

## List of abbreviations

COMT = catechol-O-methyltransferase

COMT-HH = homozygous non-polymorphic

COMT-HL = heterozygous

COMT-LL = homozygous polymorphic

SNPs = single nucleotide polymorphisms

BMI = body mass index

OR = odds ratio

CI = confidence interval

aOR = adjusted odds ratio estimate of departure from multiplicativity

## Competing interests

The author(s) declare that they have no competing interests.

## Authors' contributions

TL conceived of the study, collected genotyping samples, participated in data analysis, and drafted the manuscript. BB conducted data analysis and drafted the manuscript. JW analyzed the genotyping and drafted the manuscript. AA collected interview data from the Cape Cod population and drafted the manuscript. All authors read and approved the final manuscript.
